# A genomic atlas of systemic interindividual epigenetic variation in humans

**DOI:** 10.1186/s13059-019-1708-1

**Published:** 2019-06-03

**Authors:** Chathura J. Gunasekara, C. Anthony Scott, Eleonora Laritsky, Maria S. Baker, Harry MacKay, Jack D. Duryea, Noah J. Kessler, Garrett Hellenthal, Alexis C. Wood, Kelly R. Hodges, Manisha Gandhi, Amy B. Hair, Matt J. Silver, Sophie E. Moore, Andrew M. Prentice, Yumei Li, Rui Chen, Cristian Coarfa, Robert A. Waterland

**Affiliations:** 1 USDA/ARS Children’s Nutrition Research Center, Department of Pediatrics, Baylor College of Medicine, Houston, TX USA; 2 Department of Genetics, Evolution and Environment, UCL Genetics Institute, University College London, London, WC1E 6BT UK; 3 MRC Unit The Gambia at London School of Hygiene and Tropical Medicine, Keneba, The Gambia; 4 Department of Women and Children’s Health, King’s College London, London, UK; 5 Department of Obstetrics and Gynecology, Baylor College of Medicine, Houston, TX USA; 6 Department of Pediatrics – Neonatology, Baylor College of Medicine, Houston, TX USA; 7 Human Genome Sequencing Center, Baylor College of Medicine, Houston, TX USA; 8 Department of Molecular and Human Genetics, Baylor College of Medicine, Houston, TX USA; 9 Department of Molecular and Cellular Biology, Baylor College of Medicine, Houston, TX USA

## Abstract

**Background:**

DNA methylation is thought to be an important determinant of human phenotypic variation, but its inherent cell type specificity has impeded progress on this question. At exceptional genomic regions, interindividual variation in DNA methylation occurs systemically. Like genetic variants, systemic interindividual epigenetic variants are stable, can influence phenotype, and can be assessed in any easily biopsiable DNA sample. We describe an unbiased screen for human genomic regions at which interindividual variation in DNA methylation is not tissue-specific.

**Results:**

For each of 10 donors from the NIH Genotype-Tissue Expression (GTEx) program, CpG methylation is measured by deep whole-genome bisulfite sequencing of genomic DNA from tissues representing the three germ layer lineages: thyroid (endoderm), heart (mesoderm), and brain (ectoderm). We develop a computational algorithm to identify genomic regions at which interindividual variation in DNA methylation is consistent across all three lineages. This approach identifies 9926 correlated regions of systemic interindividual variation (CoRSIVs). These regions, comprising just 0.1% of the human genome, are inter-correlated over long genomic distances, associated with transposable elements and subtelomeric regions, conserved across diverse human ethnic groups, sensitive to periconceptional environment, and associated with genes implicated in a broad range of human disorders and phenotypes. CoRSIV methylation in one tissue can predict expression of associated genes in other tissues.

**Conclusions:**

In addition to charting a previously unexplored molecular level of human individuality, this atlas of human CoRSIVs provides a resource for future population-based investigations into how interindividual epigenetic variation modulates risk of disease.

**Electronic supplementary material:**

The online version of this article (10.1186/s13059-019-1708-1) contains supplementary material, which is available to authorized users.

## Background

Methylation of cytosines in CpG dinucleotides is an epigenetic mechanism with essential roles in mammalian development [1, 2]. To explore its functions in cellular differentiation, unbiased analysis of CpG methylation by whole genome bisulfite sequencing (WGBS) has been used to characterize epigenetic differences among different human tissues and cell types [3, 4]. Meanwhile, human interindividual variation in DNA methylation that is not cell type-specific has attracted relatively little attention. Systemic interindividual epigenetic variation is important, however, because like genetic variation it is a potential determinant of phenotype and can be assessed in any easily biopsiable DNA sample. Hence, though not the only type of epigenetic variation that might contribute to disease, systemic interindividual variants are highly advantageous for population studies. Moreover, because systemic epigenetic variants originate in the early embryo [5], their establishment can be influenced by periconceptional environment [6, 7].

**Fig. 1:**
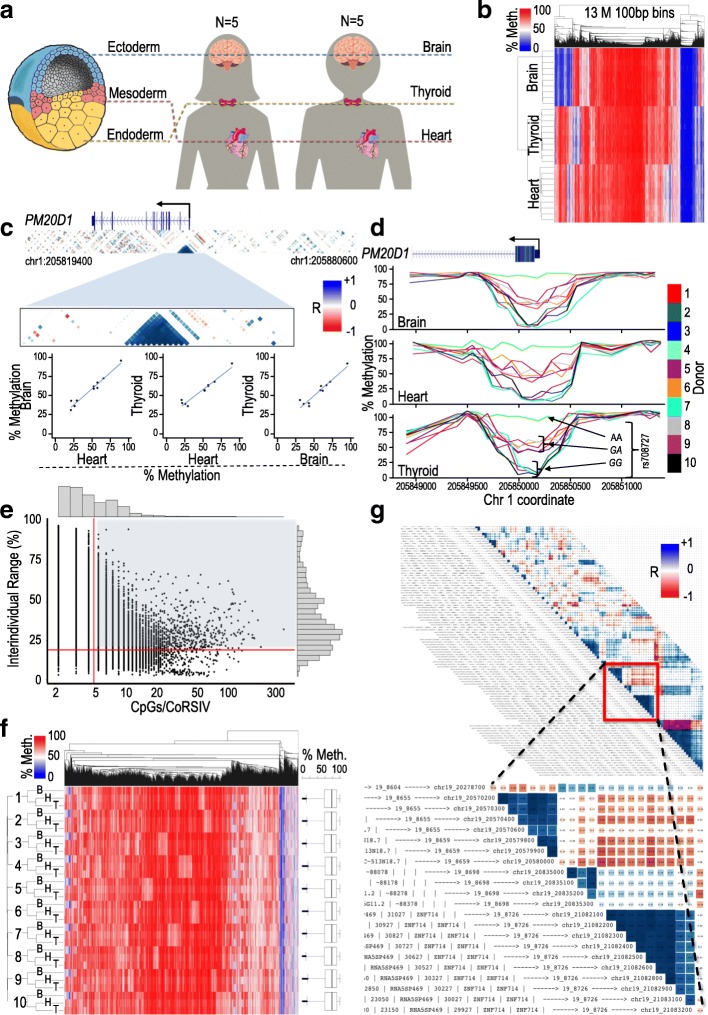
Strategy for identifying correlated regions of systemic interindividual variation (CoRSIVs). **a** The tissues analyzed represent the three germ layer lineages; 10 Caucasian GTEx donors were studied, yielding 30 methylomes. **b** Initial unsupervised clustering of whole-genome bisulfite sequencing data; considering all informative bins, they cluster by tissue. **c** Example of a CoRSIV identified at the *PM20D1* promoter. The blue triangle shows a region of correlated methylation comprising thirteen 100-bp bins; the three scatter plots illustrate its high inter-tissue correlation. **d** Plots of individual methylation at the *PM20D1* CoRSIV illustrate systemic interindividual variation. Genotype data at *rs708727* (bottom panel) indicate strong mQTL at the locus. **e** Scatter plot of interindividual methylation range vs. number of CpGs per CoRSIV, for all 39,424 CoRSIVs initially identified. Subsequent analyses focus on the 9926 CoRSIVs with ≥ 5 CpGs/CoRSIV and IIR ≥ 20 (shaded area). **f** Unlike genome-wide bins, the 9926 CoRSIV bins cluster by individual (B, H, T—brain, heart, thyroid). Box plots on right show that the 10 donors show no individual differences in average methylation across all the CoRSIVs. **g** An illustrative region from the CoRSIV plot of chr19. Inset shows example of annotation of a CoRSIV (chr19_8726) at *ZNF714*

Previous studies identified systemic interindividual variation (SIV) in DNA methylation by genome-scale DNA methylation profiling in multiple tissues from multiple individuals [5, 6, 8], but were limited by the profiling technique and/or the number of tissues and individuals studied. Here, we performed unbiased genomewide DNA methylation analysis in post-mortem thyroid, heart, and brain (representing all three germ layer lineages) from each of 10 donors in the NIH Genotype-Tissue Expression (GTEx) program [9] (Fig. 1a).

## Results

### Screen for systemic interindividual variation in DNA methylation

We performed deep WGBS on all 30 samples, generating ~ 1.2B 150-bp paired-end reads (~ 122 Gb uniquely mapped sequence per sample), yielding average genome-wide read depth of ~ 40× (Additional file 1: Table S1). Genetic identity of the three libraries representing each individual was verified by calling single nucleotide variant (SNV) genotypes from the WGBS data (Additional file 4: Figure S1). We analyzed CpG methylation at 100-bp resolution, focusing on all 100-bp human autosomal bins containing at least one CpG site and yielding adequate read depth (see Additional file 3 for methods). Considering all (~ 13M) such informative bins, genomic CpG methylation in the 30 samples clustered by tissue (Fig. 1b). Since combined effects across multiple neighboring CpGs are both more stable and more likely to affect gene expression [10], our goal was to identify regions of SIV. To maximize genomic coverage, we applied a two-stage approach. First, all reads from each individual were combined to calculate individual-level average methylation for each bin, and individually correlated regions of methylation (bin-bin *R* ≥ 0.71 (*R*^2^ ≥ 0.5)) were built in a step-wise fashion [11, 12] (see “Methods” and Additional file 3: Supplementary Methods). Then, for each such region, inter-tissue correlation (ITC) of average methylation was assessed across all tissue-type pairs. By this approach, 90% of all human autosomal bins were informative. (Sex chromosomes were not evaluated due to the limited sample size.) Regions yielding a minimum ITC ≥ 0.71 were identified as “correlated regions of SIV” (CoRSIVs) (Fig. 1c, d).

Permutation testing showed that only 39,424 (9%) of these 446,665 regions satisfy the minimum ITC criterion by chance; hence, the vast majority of the 39,424 CoRSIVs (Additional file 2: Table S2) are statistically significant. Many, however, include only a few CpGs, and some showed relatively minor interindividual variation (Fig. 1e). To target the most robust epigenetic variants likely to have the greatest biological relevance, we focused all subsequent analyses (and the remainder of this report) on the 9926 CoRSIVs containing at least 5 CpGs (Additional file 4: Figure S2a, b) and exhibiting an inter-individual methylation range of at least 20% [6, 13] (Fig. 1e; annotated list in Additional file 1: Table S3). Importantly, analysis of our permutation results showed that each of these is statistically significant (*P* < 0.05) (Additional file 4: Figure S2c). Unlike at genome-wide bins (Fig. 1b), methylation at these 9926 CoRSIVs clustered by individual (Fig. 1f). Notably, although our cutoff for minimum ITC was 0.71, most of the 9926 CoRSIVs exhibit minimum inter-tissue *R* > 0.85 (Additional file 4: Figure S2d). Analysis of DNA methylation by bisulfite pyrosequencing in an independent set of Vietnamese cadaver tissues (liver, kidney, and brain from 17 individuals) validated systemic interindividual variation at 9 of 11 loci examined (Additional file 4: Figure S3, Additional file 1: Table S4). The two that did not validate showed low interindividual variation in the Vietnamese, which might arise from chance similarities in the small sample size studied. Previous studies have estimated that 15–20% of CpG sites in the human genome show tissue-specific interindividual variation [4, 14]. CoRSIVs are exceedingly rare by comparison, comprising only ~ 0.1% of the human genome.

Since thyroid, heart, and brain do contain some cell types in common (white blood cells, for example) one might suppose that CoRSIV signals could arise from interindividual variation in the proportional representation of these contaminating cell types. Given the very small proportion of blood cells in the three tissues, it is difficult to imagine that they could explain inter-tissue *R*^2^ ≥ 0.50. Nonetheless, we devised several tests of this alternative explanation. First, analyzing publicly available WGBS data on monocytes isolated from six individuals, we show interindividual variation at CoRSIV but not control regions (Additional file 4: Figure S4), demonstrating that the interindividual variation is evident even in a single highly purified cell type. Second, using samples collected as part of a study of infant twins, we performed bisulfite pyrosequencing at several CoRSIVs in 20–30 individuals and found that interindividual variation in fingernail DNA is correlated with that in peripheral blood (Additional file 4: Figure S5). Since fingernails are composed of keratinized nail matrix cells (and contain no leukocytes) these correlations clearly are not the result of blood contamination. Lastly, we used data from the Blueprint epigenome project [15] to assess the overlap of CoRSIVs with differentially methylated regions (DMRs) distinguishing two major leukocyte subtypes: B cells and neutrophils. If CoRSIV signals result from individual variation in the proportionality of contaminating leukocytes, CoRSIVs should overlap extensively with these DMRs. However, only 37 such overlaps were found (i.e., < 1% of the 9926 CoRSIVs) (see Additional file 3 for methods). The single explanation consistent with all of these observations is that CoRSIVs are bona fide regions of systemic interindividual variation in DNA methylation. Indeed, CoRSIVs are highly enriched for CpG sites previously shown to be highly correlated across four brain regions and blood [16] (odds ratio 16.5, *P* = 1.2 × 10^− 81^) (see Additional file 3 for methods).

### Characteristics of CoRSIVs

Systemic individual differences in global DNA methylation could be attributed to genetic variants in, for example, genes regulating one-carbon metabolism or DNA methylation enzymes [17] or to technical variation in, for example, the time between death and tissue collection (post-mortem interval). In selecting donors we attempted to minimize potential sources of variation including age, body mass index, and post-mortem interval (Additional file 1: Table S5). Average CoRSIV methylation did not differ among the 10 donors studied (Fig. 1f and Additional file 4: Figure S6a), arguing against global regulation of or systematic influences on methylation at these regions. To visualize CoRSIVs throughout the genome, we generated an atlas of annotated CoRSIV maps for each human autosome (https://corsiv.shinyapps.io/CoRSIV_Plotter/) (example region shown in Fig. 1g). A striking feature of these maps is the extensive long-range correlation (and anti-correlation) among CoRSIVs. Long-range interindividual correlation in DNA methylation was previously reported in population studies of peripheral blood methylation using a commercial methylation array [12, 18]. For example, two regions separated by ~ 20 kb and overlapping the 5′ and 3′ ends of *SPATC1L*, previously identified as regions of anticorrelated methylation in peripheral blood [12], are in fact anticorrelated CoRSIVs (Additional file 4: Figure S7a). Since most haplotype blocks in Caucasians are < 50 kb in length [19], we were surprised to observe many examples of positively intercorrelated CoRSIV pairs spanning much larger genomic distances, which we refer to as “superCoRSIVs” (Additional file 4: Figure S2f). Topologically associated domains (TADs) are broad genomic regions (median size ~ 1 Mb) with a high probability of physical association and are largely invariant across different cell types [20]. We therefore tested whether superCoRSIVs tend to occur within TADs; indeed, across 10 human tissues, the proportion of superCoRSIVs wholly within TADs was consistently elevated relative to that of a set of matched control regions (paired *t*-test *P* = 10^− 5^, Additional file 4: Figure S2f). Since TAD boundaries are associated with CTCF sites [20], our observation that CTCF binding sites are enriched within SuperCoRSIVs (*χ*^2^ test *P* = 0.003, Additional file 4: Figure S2 g) additionally suggests a mechanistic link between SuperCoRSIVs and TADs.

**Fig. 2:**
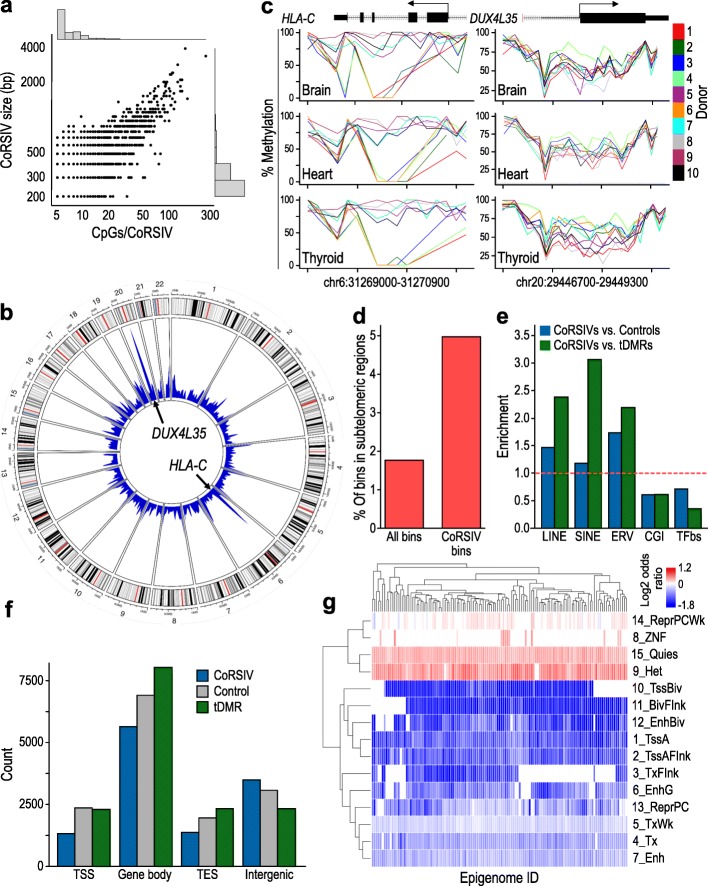
Distribution and characteristics of 9926 human CoRSIVs. **a** Scatter plot of CoRSIV size vs. CpGs per CoRSIV. **b** Circos plot of human autosomes indicates regions of high CoRSIV density. **c** Individual methylation levels across examples of CoRSIVs from each of the two most high-density regions: (left) *HLA-C* (chromosome 6) and (right) *DUX4L35* (chromosome 20). **d** Compared to all informative bins, CoRSIVs are more than twofold enriched in sub-telomeric regions (*χ*^2^ test *P* < 10^− 300^). **e** Compared to control regions or tissue-specific differentially methylated regions (tDMRs), CoRSIVs are enriched for repetitive elements and depleted for CpG islands (CGI) and transcription binding sites (TFbs) (*χ*^2^ test *P* < 10^− 8^ for all comparisons). **f** Relative to control and tDMR regions, CoRSIVs are under-represented at transcription start sites (TSS), within gene bodies, and at transcription end sites (TES) (*χ*^2^ test *P* < 10^− 16^ for all comparisons). **g** ChromHMM epigenomic features significantly enriched or depleted in CoRSIVs

Whereas most of the 9926 CoRSIVs are only 200–300-bp long and include 5–10 CpGs, the largest span several kb and involve hundreds of CpGs (Fig. 2a). Rather than being randomly distributed throughout the genome, CoRSIVs tend to occur in clusters (Kolmogorov–Smirnov test vs. uniform distribution: *P* < 10^− 100^). The two biggest peaks of CoRSIV density are observed at the major histocompatibility (MHC) locus on chromosome 6 and the pericentromeric region on the long arm of chromosome 20 (Fig. 2b); illustrative profiles of individual methylation at one gene from each region are shown in Fig. 2c. SIV in DNA methylation was previously reported in the MHC locus [21]. We are not aware of previous publications, however, highlighting the exceptional epigenetic behavior of the chromosome 20 region. Interestingly, whereas the 92 CoRSIVs spanning the MHC region are largely independent of one another (Additional file 4: Figure S8a,b), the 56 CoRSIVs comprising the chromosome 20 region are highly intercorrelated (Additional file 4: Figure S8c,d), indicating that individual methylation status is correlated across this entire ~ 2.3-Mb region. As suggested by the circos plot (Fig. 2b) and the higher-resolution plots in Additional file 4: Figure S9, CoRSIVs are > 2-fold enriched in subtelomeric regions (*χ*^2^ test *P* < 10^− 300^, Fig. 2d, Additional file 1: Table S6). Given that subtelomeric regions are highly variable, one might suppose that the CoRSIV enrichments in these regions might be due to poor mapping rates leading to artifacts. However, unique mapping rates in the chromosome 20 pericentromeric region were generally above, and those in subtelomeric regions only slightly below, the genomewide average (Additional file 4: Figure S10). Further, only 14 of the 9926 CoRSIVs overlapped an ENCODE blacklist region [22] (genomic regions known to yield artifacts in functional genomics studies) (see Additional file 1: Table S19).

To explore sequence features associated with CoRSIVs, we generated two reference sets of genomic regions: a randomly selected set of genomic regions matched to the CoRSIVs on chromosome, size, and CpG content (“controls”) (Additional file 4: Figure S11a, Additional file 1: Table S7), and a set of similarly matched loci drawn from regions of tissue-specific differential methylation (tDMRs) (Additional file 4: Figure S11b,c, Additional file 1: Table S8). Compared to both reference sets, CoRSIVs are enriched for transposable elements and depleted for CpG islands (CGI) and transcription factor binding sites (*χ*^2^ test *P* < 10^− 8^ for all comparisons; Fig. 2e, Additional file 4: Figure S2 h, Additional file 1: Table S9). Relative to either controls or tDMRs, CoRSIVs are under-represented within and near genes and enriched in intergenic regions (*χ*^2^ test *P* < 10^− 16^; Fig. 2f, Additional file 1: Table S10). Analysis of ChromHMM features across 111 reference human epigenomes (derived from the NIH Epigenome Roadmap project) [23] shows that, relative to control regions, CoRSIVs are enriched in quiescent regions and those associated with repressive polycomb marks and depleted in heterochromatic regions, active promoters, and enhancers (Fig. 2g); the strongest depletions were found for promoter and enhancer regions characterized as “bivalent” (i.e., poised between active and inactive states) in embryonic stem cells [24]. Similar depletions/enrichments are observed when CoRSIVs are compared to tDMRs (Additional file 4: Figure S2e, Additional file 1: Tables S11, S12). Analysis of data on genomic evolutionary rate profiling scores [25] showed that, relative to both control and tDMR regions, CoRSIVs tend not to occur in highly conserved genomic regions (CoRSIVs vs. controls odds ratio = 0.4, *P* = 8.2 × 10^− 118^; CoRSIVs vs. tDMRs odds ratio = 0.2, *P* < 1.0 × 10^− 200^) (Additional file 4: Figure S2i).

### Influence of genetic variation on CoRSIVs

**Fig. 3:**
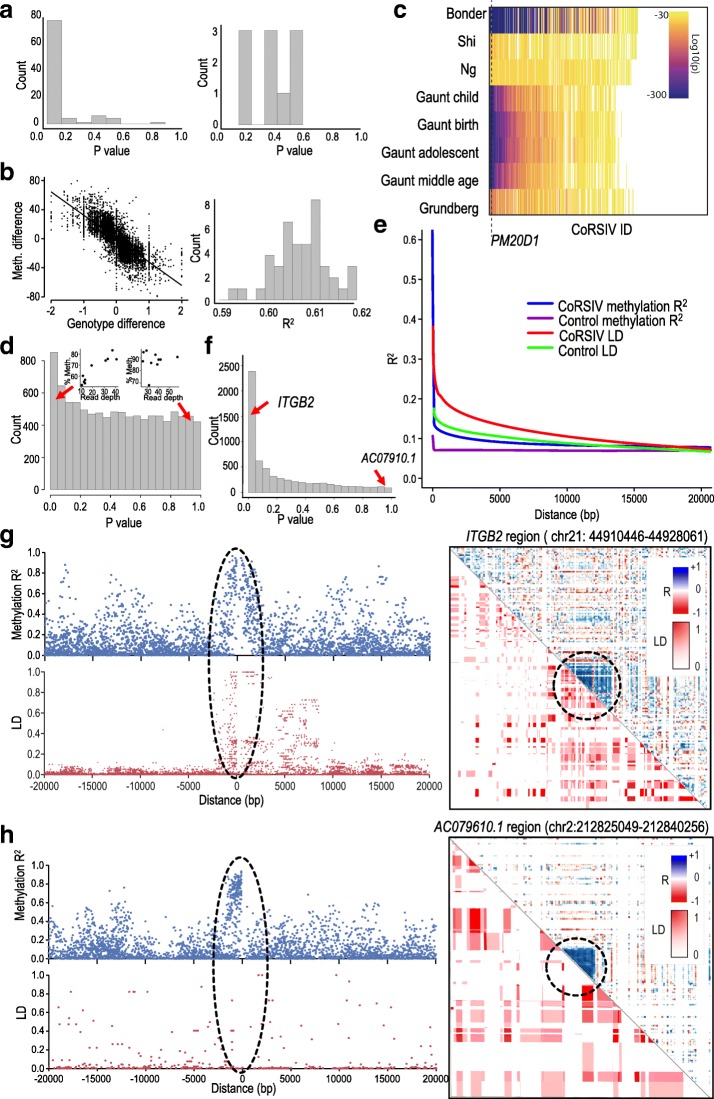
Evaluating associations between CoRSIV DNA methylation and neighboring genetic variation. **a** CoRSIV-specific *P* value distributions from mQTL analyses at the two most CoRSIV-dense genomic regions. At the MHC locus on chromosome 6, most CoRSIVs show mQTL; none of 10 informative CoRSIVs in the pericentromeric region on chromosome 20 show mQTL. **b** Summary of pair-wise analyses. Scatter plot shows correlation of interindividual differences in CoRSIV methylation vs. interindividual differences in average CoRSIV-associated SNV genotype for one pair of GTEx donors (RVPV and X261). Distribution of all such pairwise comparisons (right); average *R*^2^ = 0.61. **c** Heat map of published mQTL effects at 819 CoRSIVs overlapping probes on the HM450 array. Approximately 40% (white) show no evidence of mQTL. Dotted line shows location of probes within the *PM20D1* CoRSIV. **d** Histogram of *P* values for the spearman correlation between methylation and read coverage within each CoRSIV. Two insets above the histogram show two scatter plots drawn from opposite ends of the histogram. The top left inset shows a strong association (*P* < 0.05) between average methylation in chr3_2398 CoRSIV and individual-average read depth in this region. The top right inset shows weaker association (*P* > 0.85) between average methylation in chr6_24299 CoRSIV and individual-average read depth in this region. **e** Decay curves of correlation in methylation and LD for CoRSIVs and control regions. **f** Histogram of CoRSIV-specific *P* values for the Spearman correlation analyses of methylation decay vs. LD decay. Locations of two CoRSIVs with (*ITGB2*) and without a significant association (*AC07910.1*) are indicated. **g** The CoRSIV at *ITGB2* yielded a significant association (*P* < 0.05) between methylation decay and LD decay, consistent with a haplotype-effect on methylation. The scatter plot on the left (top, blue) shows all methylation *R*^2^ for pairs of bins in which one bin is within the CoRSIV and other bin is within ± 20 kb. The scatter plot on the left (bottom, red) shows LD for all pairs of SNVs. For each pair one SNV is within the CoRSIV and the other is within 20kb. On the right, the upper diagonal shows the CoRSIV (blue) in this region, and the lower diagonal shows the LD pattern in this region (1000 Genomes CEU). **h** In contrast, the CoRSIV at *AC07910.1* showed no association between methylation decay and LD decay (*P* > 0.9), suggesting pure epigenetic variation

We took several complementary approaches to evaluate the extent to which DNA methylation at CoRSIVs is genetically determined. Associations between genetic variation and DNA methylation can be assessed relative to single genetic variants (methylation quantitative trait loci—mQTL) [26, 27] or at the haplotype level (haplotype-dependent allele-specific methylation) [28]. As previously reported [29], the *PM20D1* promoter region illustrated in Fig. 1d exhibited strong mQTL. With methylation data on only 10 individuals, however, we were underpowered to analyze mQTL at all 9926 CoRSIVs, so performed focused analyses on several regions of high CoRSIV density (Fig. 2b). We used donor-specific SNV data from GTEx [9, 30] to test for associations between individual-average CoRSIV methylation and SNV genotype. Since the strongest mQTL effects occur over fairly short distances [28], we assessed associations with common variants within 10 kb of each CoRSIV and, to minimize false positives, considered only SNVs for which all three genotypes were represented. Of 96 informative CoRSIVs in the high-density region overlapping the MHC locus on chromosome 6 (Fig. 2b, Additional file 4: Figure S8a), 74 showed significant mQTL (Fig. 3a, left), corroborating a previous report that the MHC locus is an mQTL hotspot [12]. Conversely, in the high-density CoRSIV region near the chromosome 20 centromere (Fig. 2b, Additional file 4: Figure S8c), none of 10 informative CoRSIVs showed mQTL (Fig. 3a, right). Next, we considered all pairwise comparisons among the 10 donors to test associations between interindividual differences in methylation and average SNV genotype in the 10-kb regions flanking each CoRSIV. This analysis (Fig. 3b) indicated that ~ 60% of interindividual variation in CoRSIV methylation is explained by genetic variation in *cis*. We also exploited several published mQTL data sets based on the Illumina HM450 array [26, 27, 31–33]. Of the ~ 485,000 CpG-specific probes included on the HM450 array, just 1659 overlap 819 CoRSIVs (Additional file 1: Table S13). Across eight populations studied by five different groups, approximately half of these CoRSIVs consistently showed mQTL, while ~ 40% showed no evidence of mQTL (Fig. 3c, Additional file 1: Table S14). The methylation distribution in each of the 10 GTEx donors is substantially different for mQTL-negative vs. mQTL-positive CpGs (Additional file 4: Figure S6b), providing independent corroboration of the two classes evident in the HM450 mQTL analysis.

We next used the DECIPHER database [34] to test whether CoRSIVs might be explained by copy number variations (CNVs). Unsurprisingly (given their association with subtelomeric regions), CoRSIVs were significantly enriched for overlaps with CNV regions compared to control (odds ratio = 1.8, *P* = 1.7 × 10^− 94^) and tDMR regions (odds ratio = 2.7, *P* = 8.1 × 10^− 100^). To determine if SIV among the 10 GTEx donors is associated with deletions or duplications, we calculated Spearman correlations between average WGBS read depth and average CoRSIV methylation across the 10 individuals. The *P* value distribution (Fig. 3d) shows that, for the vast majority of CoRSIVs, individual-average methylation is not significantly associated with read depth, indicating that CNV generally does not explain SIV at CoRSIVs.

Previous population-based studies have examined haplotype effects on regional methylation by comparing the decay rate of correlation in methylation with that of genetic linkage [11, 12]. We likewise analyzed methylation data at CoRSIVs in the context of linkage disequilibrium (LD) data on Caucasian individuals (CEU) in the 1000 genomes project [35]. Relative to control regions, CoRSIVs were associated with regions of higher LD, and methylation in CoRSIVs was more highly correlated with that in flanking regions (Fig. 3e). Whereas previous studies on methylation variants in Arabidopsis [11] and humans [12] found that methylation correlation decays very rapidly (within 1–2 kb), methylation decay at CoRSIVs is much longer, not returning to baseline until ~ 20 kb (Fig. 3e). To examine regional associations between genetic and methylation variation, we therefore assessed the linear correlation between methylation decay and LD decay within 20 kb upstream and downstream of each CoRSIV. The resulting *P* value distribution (Fig. 3f, Additional file 1: Table S15) is highly enriched for significant values, providing evidence of genetic influence on methylation. Examples drawn from opposite ends of the *P* value distribution illustrate that this approach distinguishes CoRSIVs located entirely within haplotype blocks—and putatively under genetic control—(Fig. 3g) from those in which strong regional correlation in methylation occurs in the absence of underlying haplotype structure (Fig. 3h), indicating “pure” epigenetic variation [11].

Together, these analyses show that methylation at many CoRSIVs is influenced by genetic variation in *cis* but also suggest that, at some of these loci, systemic interindividual epigenetic variation may be independent of genetic variation. With the data currently available, we cannot yet make definitive conclusions about the absence of genetic effects. Characterization of all possible genetic influences on CoRSIV methylation (including *trans* effects, which were not evaluated here) will require methylation analyses targeted to these regions in a large number of genotyped individuals. Systemic interindividual epigenetic variation that is largely independent of genetic variation is consistent with metastable epialleles [36]. In mouse and human studies, epigenetically metastable loci are characterized by stochastic interindividual variation in epigenotype that is established in the early embryo [5] and influenced by periconceptional environment [7, 8, 37, 38].

### Influence of periconceptional environment on CoRSIVs

**Fig. 4:**
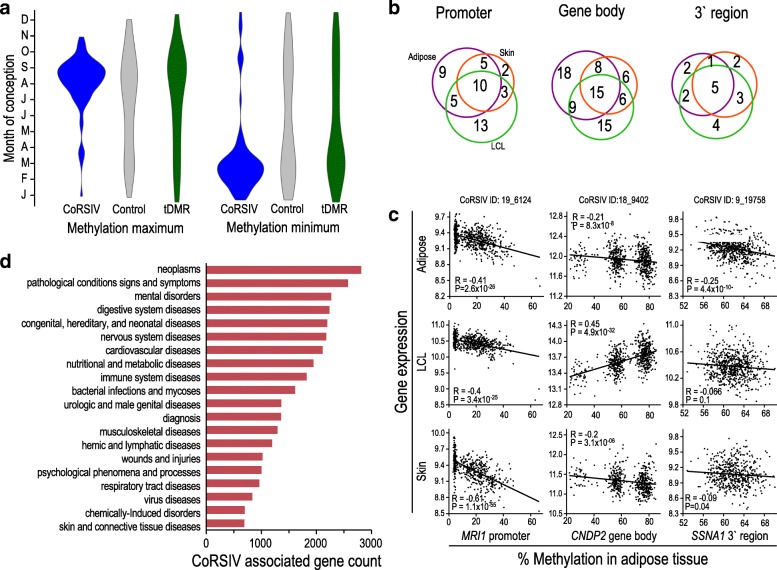
CoRSIVs show influence of periconceptional environment, correlate with gene expression, and are associated with human disease. **a** Seasonal variation of blood methylation at CoRSIVs, controls, and tDMRs in 233 Gambian 2-year olds [13]. Data represent CpGs showing significant seasonal variation (FDR < 20%, see “Methods”). Predicted methylation maxima for CpGs within CoRSIVs occur in conceptions at the peak of the Gambian rainy season (July–September); minima fall within the Gambian dry season (Jan–April). Seasonal patterns at control regions and tDMRs are less pronounced (Fisher’s exact test *P* > 0.8). **b** Results of analysis linking gene expression in adipose tissue, skin, and lymphoblastoid cell lines (LCL) vs. methylation in adipose tissue [26] for 645 gene-associated CoRSIVs that are informative on the HM450 array. Venn diagrams show that for most genes that show a significant association (Spearman *P* < 0.05) between methylation and expression in adipose tissue, methylation in adipose tissue is also associated with expression in skin or LCL. **c** Examples of expression vs. CoRSIV methylation data in all three tissues at the *MRI1* promoter (left), the *CNDP2* gene body (middle), and the 3′ end of *SSNA1* (right). **d** Summary of an automated PubMed literature-search using PubTator. Shown are MESH code labels corresponding to the top 100 human diseases linked to CoRSIV-associated genes

To assess potential epigenetic metastability at these loci, we examined data from a natural experiment in a well-characterized population of subsistence farmers in rural Gambia in which annual climatic variation causes dramatic seasonal variation in maternal nutritional status and energy balance. Previous studies in this population show that, at candidate and bona fide metastable epialleles, children conceived during the peak of the rainy season show persistent and systemic elevations in DNA methylation compared to children conceived during the dry season, and these correlate with the mothers’ methyl donor metabolomes assessed in early gestation [37]. Here, rather than focus on pre-selected epochs, we studied 233 children conceived throughout the calendar year [13] and performed Fourier regression analysis to determine seasonal effects agnostically. DNA methylation in peripheral blood was measured by Illumina HM450 arrays, so this analysis is limited to CoRSIVs and control regions that are informative on this platform. Significant seasonal variation was highly enriched at CoRSIVs (Fisher’s exact test *P* = 1.7 × 10^− 6^) but not at controls or tDMRs (Fisher’s exact test *P* = 1.0 and 0.85, respectively, Additional file 4: Figure S12a,b). Moreover, this unsupervised analysis independently validated seasonal effects corresponding to peaks of the rainy (July–September) and dry (January–April) seasons (Fig. 4a) consistent with previous studies of human metastable epialleles [6, 8, 13, 37]. It should be noted that in these outbred human populations we have yet to rule out potential confounding by genetic variants that influence both season-specific fecundity and systemic methylation. This caveat notwithstanding, these data provide independent evidence that a significant proportion of CoRSIVs exhibit epigenetic metastability, which can be influenced by maternal nutrient status during early pregnancy.

### Associations of CoRSIVs with gene expression and human disease

CoRSIVs show great promise for epigenetic epidemiology [39] because, at these loci, DNA methylation measurements in any easily biopsied tissue should provide information about epigenetic regulation throughout the body. To test this, we analyzed data from a previous large population study of adult women in which DNA methylation in adipose tissue was measured by the HM450 array and gene expression was assessed in adipose tissue, skin, and lymphoblastoid cell lines [26]. Unlike at control regions and tDMRs (Additional file 4: Figure S13b), among gene-associated CoRSIVs that showed an association between methylation and expression in adipose tissue, most also showed an association between methylation in adipose tissue and gene expression in skin or lymphoblastoid cell lines (Fig. 4b,c, Additional file 4: Figure S7b, Additional file 1: Table S16). These data substantiate the practical utility of CoRSIVs, in that non-invasive measurements of DNA methylation (for example, in peripheral blood) can provide an indication of epigenetic regulation in other tissues. To explore this further, we analyzed a recently published database encompassing 1319 “epigenome-wide association studies” (EWASdb) [40] (based on DNA methylation, many in peripheral blood). As expected (based on the systemic nature of CoRSIVs), this analysis showed that disease-associated CpG sites are 37% enriched in CoRSIVs relative to control regions (*χ*^2^ test *P* = 2.2 × 10^− 4^) and 53% enriched in CoRSIVs relative to tDMRs (*χ*^2^ test *P* = 6.5 × 10^− 8^) (Additional file 4: Figure S12e). To explore these associations in greater depth, we began by dividing the disease outcomes into cancer and non-cancer diseases, since the HM450 array is enriched for CpG sites known to be hypermethylated in cancer, and many epigenetic studies of cancer are based on comparing tumor vs. normal tissue, which is not relevant to the systemic nature of CoRSIVs. Whereas CoRSIV probes were not enriched for disease associations with cancer (Additional file 4: Figure S14), they did show strong associations to non-cancer diseases (Additional file 4: Figure S15). The greatest enrichments were found for “deletion or duplication of chromosome 7q11.23,” “immunodeficiency, centromeric instability and facial abnormalities (ICF) syndrome,” “asthma,” “down syndrome,” and “human immunodeficiency virus.” Notably, though several of these relate to immune function, none of the probes driving these enrichments were within the CoRSIV-rich MHC region on chromosome 6.

Although CoRSIVs are depleted in and around genes (Fig. 2f), there are thousands of CoRSIV-associated genes. As another way to explore potential phenotypic consequences of epigenetic variation at CoRSIVs, we used GREAT [41] to perform gene ontology analysis (Additional file 4: Figure S16). The main significant enrichments (vs. genomic background) relate to the high density of CoRSIVs identified in the MHC locus. Otherwise, CoRSIVs were not associated with specific cellular processes or components. We used an automated literature search tool (PubTator) [42] to search PubMed and identify diseases linked to the 3127 CoRSIV-associated genes. This analysis initially found that, compared to all genes listed in PubMed, CoRSIV-associated genes are enriched for associations with hundreds of diseases, particularly those involving the brain (Additional file 1: Tables S17, S18, Additional file 4: Figure S12c). Recent studies have shown that genes expressed in the brain (particularly in neurons) tend to be longer than average [43], so we tested whether our analysis based on gene-body overlap could bias toward longer genes. Indeed, relative to all PubTator genes, CoRSIV-associated genes are enriched for long genes (Additional file 4: Figure S12d). After adjusting for gene length, CoRSIV-associated genes were no longer associated with diseases involving the brain (Additional file 1: Table S17). Nonetheless, thousands of CoRSIV-associated genes are implicated in a wide range of human diseases including neoplasms, mental disorders, digestive, nervous system, and cardiovascular diseases (Fig. 4d, Additional file 1: Table S18). Recent reports highlight several examples, including *SPATC1L* in male infertility [44], *PM20D1* in Alzheimer’s disease [29], and *DUSP22* in lymphoma [45].

## Discussion

Here, we have uncovered, charted, and characterized a previously unrecognized level of molecular individuality in humans. Although CoRSIVs were identified in Caucasians, we validated systemic interindividual epigenetic variation at these loci in an Asian cohort and confirmed interindividual variation and documented an influence of periconceptional environment in rural Africans. Hence, together our data indicate that CoRSIVs are an ancestral and universal feature of the human genome.

Our study is not without limitations. Since our screen included only 10 individuals, all Caucasian, this first CoRSIV atlas is incomplete. For example, we did not detect a known CoRSIV at *VTRNA2-1* [6, 46] because all 10 individuals happen to exhibit normal (~ 50%) methylation at the locus. Another potential caveat (which extends to most studies using GTEx samples) is that many of the donors studied were at late stages of disease (Additional file 1: Table S5). Future screens surveying additional individuals (and diverse ethnic groups) will undoubtedly identify additional human CoRSIVs. Lastly, our use of the term “systemic” merits clarification. Our intended meaning is that systemic epigenetic variants are generally consistent across all tissues and cell types. Indeed, although our screen was based on thyroid, heart, and brain, our various validation studies additionally confirmed interindividual variation in liver, kidney, peripheral blood, and fingernail. Nonetheless, given the ~ 200 cell types in the human body, it is impossible to rule out cell-type specific effects at some of these loci. For example, it was recently shown that methylation at the *VTRNA2-1* CoRSIV lacks interindividual variation specifically in the cerebellum (but not in the cerebral cortex) [47].

Our analyses indicate that, at some of these loci, systemic interindividual epigenetic variation may not be determined by local genetic control. This somewhat surprising finding is consistent with the most powerful whole-genome analysis of genetic effects on methylation [14], which concluded that most interindividual variation in DNA methylation in human peripheral blood and adipose tissue is not associated with genetic variation. Future targeted analyses—sufficiently powered to detect both *cis* and *trans* effects—will be required to determine if a subset of CoRSIVs are, indeed, metastable epialleles. Identifying a substantial number of metastable epialleles in the human genome—including some under partial genetic influence [5, 13]—would provide support for the thesis that stochastic interindividual epigenetic variation is evolutionarily advantageous and “hard wired” into the human genome [48]. Given the ability to now use existing banked DNA samples (such as from peripheral blood) to broadly assay systemic and stable individual epigenetic variants, we anticipate that this CoRSIV atlas will accelerate future progress in the field of epigenetics and human disease [49].

## Methods

### Study populations

Tissue samples from the NIH Genotype-Tissue Expression (GTEx) program were collected during rapid autopsy or organ transplant settings, so most organs will be free of major disease processes [9]. Inclusion criteria were as follows: donor age 50–69, no morbid obesity (BMI 18.5–35), postmortem interval (death to tissue collection) < 24 h, no recent blood transfusions, no metastatic cancer, and no chemotherapy or radiation therapy in the last 2 years. The 10 Caucasian donors were balanced by sex and were selected based on the availability of all three tissues (thyroid, heart, brain).

### Analytical approach

Deep whole-genome bisulfite-sequencing (WGBS) was performed on 30 tissue samples and were preprocessed as described in Additional file 3: Supplementary methods. To maximize genomic coverage, we applied a two-stage approach to identify regions of SIV. First, all reads from each individual were combined to calculate individual-level average methylation for each bin, and individually correlated regions of methylation (bin-bin *R* ≥ 0.71 (*R*^2^ ≥ 0.5)) were built in a step-wise fashion [11, 12] (for details see Additional file 3: Supplementary Methods). Then, for each such region, inter-tissue correlation (ITC) of average methylation was assessed across all tissue-type pairs. Regions yielding a minimum ITC ≥ 0.71 were identified as “correlated regions of SIV” (CoRSIVs). Five complementary approaches were considered to evaluate the influence of genotype on CoRSIVs. The associations of CoRSIVs with many genomic features such as genes, sub-telomeric regions, repetitive elements, CpG islands, transcription factor binding sites, and periconceptional environment were evaluated. An assessment of CoRSIV-associated human gene and disease associations were conducted based on PubMed using Pubtator framework (see Additional file 3: Supplementary methods, for details).

### Statistical analysis

A permutation test was developed to evaluate the probability of CoRSIVs arising by chance. In each of 100,000 permutations, we scrambled subject IDs within each tissue type, computed ITCs for each of 1000 randomly selected blocks, and counted how many times a minimum ITC ≥ 0.71 was obtained (see Additional file 3: Supplementary methods, for details). Statistical significance of enrichments/depletions of CoRSIVs in various genomic contexts was calculated relative to two reference sets (controls, tDMRs) using *χ*^2^ tests. Associations of various epigenome states with CoRSIVs were analyzed by Fisher’s exact test. Analyses of mQTL using the genotype data from 10 GTEx donors used linear regression; CoRSIVs with regression coefficient *β* ≥ 10, *R*^2^ ≥ 0.5, and FDR < 5% were considered positive for mQTL. Associations between CoRSIV methylation and gene expression were evaluated based on the Spearman correlation, adjusted for multiple testing using the Benjamini-Hochberg method. Enrichment of seasonal effects at CoRSIVs and the two reference sets was determined using Fourier regression models and Fisher’s exact test.

## Additional files

Additional file 1: Supplementary Tables S1, S3-S19. (XLS 26942 kb)

Additional file 2: Supplementary Table S2. (CSV 31408 kb)

Additional file 3: Supplementary methods. (DOCX 359 kb)

Additional file 4: Supplementary Figures. (DOCX 15207 kb)
