# Supplementary material for: A genomic atlas of systemic interindividual epigenetic variation in humans
Source: Genome Biol. 2019 Jun 3;20:105. doi: 10.1186/s13059-019-1708-1 (PMC6545702; doi:10.1186/s13059-019-1708-1)
Supplement: Supplementary file 3 — Supplementary methods. (DOCX 359 kb) [file 13059_2019_1708_MOESM3_ESM.docx]

Supplementary methods for

**A Genomic Atlas of Systemic Interindividual Epigenetic Variation in Humans**

Chathura J. Gunasekara, C. Anthony Scott, Eleonora Laritsky, Maria S. Baker, Harry MacKay, Jack D. Duryea, Noah J. Kessler, Garrett Hellenthal, Alexis C. Wood, Kelly R. Hodges, Manisha Gandhi, Amy B. Hair, Matt J. Silver, Sophie E. Moore, Andrew M. Prentice, Yumei Li, Rui Chen, Cristian Coarfa*, Robert A. Waterland^*^

^*^Correspondence to: [waterland@bcm.edu](mailto:waterland@bcm.edu) or [coarfa@bcm.edu](mailto:coarfa@bcm.edu)

**Other Supplementary Material for this manuscript includes:**

- Supplementary Tables S1 to S19 (Except Table S2) (Additional file 1)
- Supplementary Table S2 (Additional file 2)

| **Table** | **Contents** |
| --- | --- |
| S1 | Sequencing depth of each library |
| S2 (Additional file 2) | Annotated list of all genomic bins within the 39,424 CoRSIVs (Unfiltered) |
| S3 | **Annotated list of all genomic bins within the 9,926 CoRSIVs (Filtered for number of CpGs and Interindividual Range)** |
| S4 | Summary of pyrosequencing primers and validation of assays |
| S5 | Donor Information GTeX ID and Color Scheme for figures |
| S6 | Subtelomeric enrichment of CoRSIVs |
| S7 | Control regions |
| S8 | tDMRs |
| S9 | Enrichment of CoRSIVs in transposable elements, CGI and TFbs |
| S10 | Enrichment of CoRSIVs with genes and intergenic regions |
| S11 | ChromHMM CoRSIVvsControls |
| S12 | ChromHMM CoRSIVvstDMRs |
| S13 | HM450k probes overlapping CoRSIVs |
| S14 | Existing mQTL data overlapping CoRSIVs |
| S15 | Associations between CoRSIV average methylation R-Squared and Linkage Disequlibrium (LD) |
| S16 | Associations between CoRSIV average methylation in adipose tissue and expression in adipose tissue, LCL, and skin |
| S17 | MESH Codes for gene associated CoRSIVs |
| S18 | PubTator Mining for disease association of CoRSIV genes |
| S19 | CoRSIVs overlapped with ENCODE black list regions |

- Supplementary Figures S1 to S16 (Additional File 4)

**Supplementary Methods**

GTEx Samples

Genomic DNA samples from thyroid, heart (left ventricle) and brain (cerebellum) tissues were obtained from 10 donors (5 males, 5 females, average age 59.2 years) in the NIH Genotype-Tissue Expression (GTEx) program[1] (Table S5). These tissues represent respectively the ectodermal, mesodermal, and endodermal germ layers of the early embryo.

Library Construction

The Bisulfite library was made using KAPA LTP kit (Roche) with modification. In brief, 5.8ul of Bisulfite-conversion control was added to 1ug genomic DNA, and the mixture was subject to shearing using covaris to 180-220bp in size. After purification through AMpure XP beads, end repair, and A-Tailing was carried out. 5ul of Seqcap library adaptor (Roche) was ligated to the sample, and the product with a size of 250-450bp was selected through Ampure XP beads. Bisulfite conversion was carried out on the product using EZ DNA methylation-lighting kit (Zymo Research), and the library was generated by amplification using KAPA HIFI Hotstart Uracil+ Ready Mix and Pre LM-PCR Oligo 1 & 2 (Roche).

Sequencing

Sequencing was performed at the Beijing Genomics Institute using the Illumina HighSeq4000, as per manufacturer’s instructions.

Bisulfite-Seq Mapping and Methylation Calling

We performed quality control, mapping, and methylation extraction from the 30 bisulfite sequencing libraries as previously described[2]. Sequencing reads were mapped to hg38 (GRCh38) genome build. Read-depths of the 30 libraries are available in the Table S1.

Post Alignment Quality Control

As a quality control step, we performed SNV-calling from these 30 libraries to ensure that different tissue samples were properly assigned to the same individual (i.e. no sample mixups). We used Bis-SNP to infer genotype directly from the Bisulfite-seq reads mapped to chromosome 19[3]. All pair-wise tissues were compared to count number of SNVs common in both tissues. These counts were then assembled into a matrix and were plotted using R software. The results confirmed that tissue sample triads were correctly matched to correct individual. The summary of the analysis is represented in Figure S1.

Algorithm for CoRSIV Detection


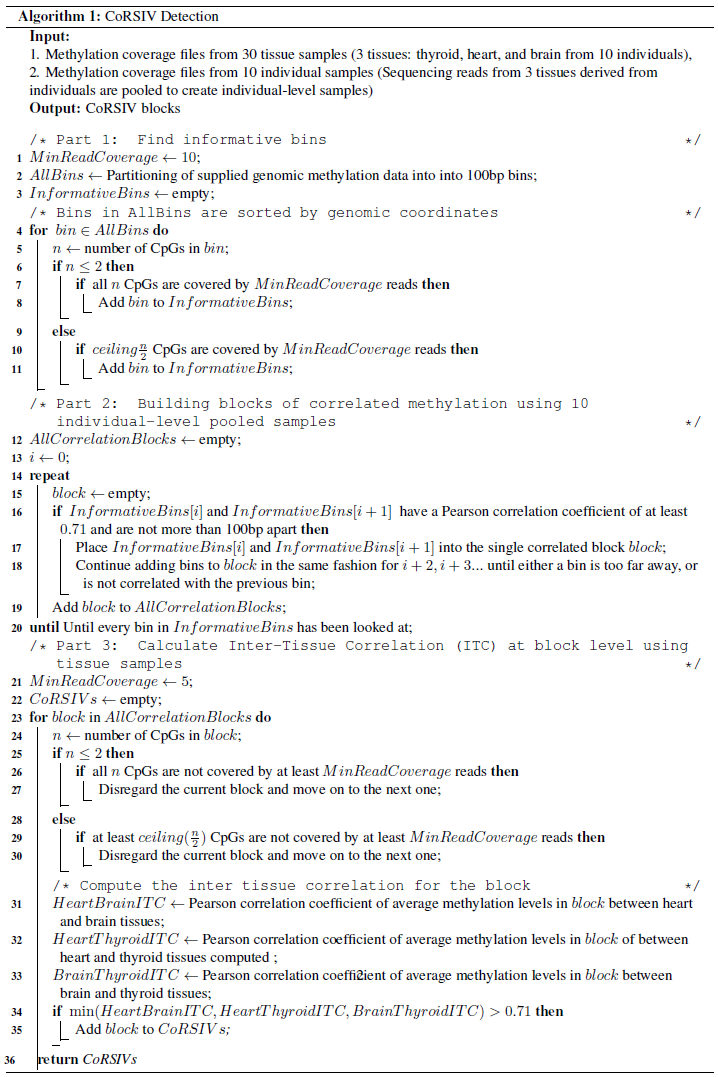


Note regarding CoRSIV screen

Some might question the use of the Pearson correlation coefficient in our screen, since our data in many cases violate assumptions such as normal distribution of lack of outliers. The assumptions of the Pearson correlation coefficient are required for hypothesis testing, i.e. testing the null hypothesis using p values[4]. Our CoRSIV screen did not use the Pearson correlation (R) for hypothesis testing. To test the statistical significance of the CoRSIVs we used permutation testing, which requires no assumptions regarding the distribution of the data. Extreme observations (outliers) do affect the Pearson correlation coefficient. But the goal of our CoRSIV screen is to detect variation, so minimizing outliers using a Spearman (rank based) test would be counterproductive. Also, our screen is naturally resistant to artifacts, because bins yielding extreme values in only one tissue are filtered out. Extreme values that are consistent across three independent measurements are not outliers, but indicate an individual variant.

Permutation Test for CoRSIVs

To test the statistical significance of CoRSIVs, we performed a permutation test to scramble subject IDs for each tissue library.

1. Begin with blocks that were identified in the step 3 of the algorithm for CoRSIV detection.
2. Repeat the following steps 100,000 times:
   1. Randomly draw 1000 blocks
   2. Permute the 10 individual IDs within 3 tissues
   3. Compute minimum ITC for the 1000 blocks
   4. Separate blocks into two groups by m.ITC >= 0.71 (A) or m.ITC < 0.71 (B) while keeping track of the block ID


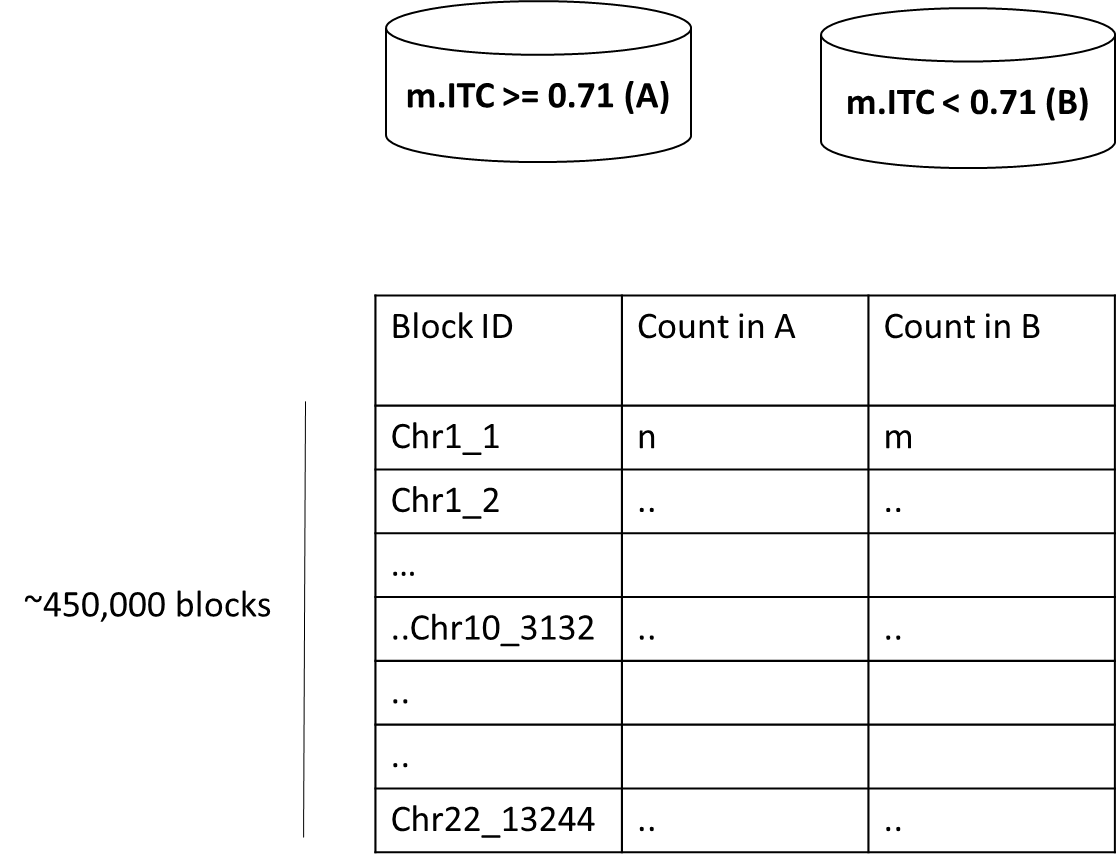


1. Compute the number of CpG, number of bins, interindividual range (IIR)
   1. Number of CpGs in a block (nCpGs)
      - nCpGs=2, A/(A+B)
      - nCpGs=3, A/(A+B)
      - ….
      - Where A = number of blocks which m.ITC >= 0.71, B=m.ITC<0.71
   2. Number of bins in a block (nBins)

- nBins = 2, A/(A+B)
- nBins =3, A/(A+B)
- …..
- Where A = number of blocks which m.ITC >= 0.71, B=m.ITC<0.71
  1. Interindividual methylation range (IIR)
     - For each IIR values in original data and get the A/(A+B)

Bisulfite pyrosequencing in 17 Asian samples (liver,kidney,brain)

Quantitative analysis of selected CoRSIVs was performed by bisulfite pyrosequencing[5] across endodermal (liver), mesodermal (kidney), and ectodermal (brain) tissue in 17 Asian cadavers[6]. Prior to use, all pyrosequencing assays were validated for linearity and sensitivity using human genomic methylation standards[6, 7] (Table S4). For each pyrosequencing assay methylation was averaged across multiple CpG sites for each sample, and inter-tissue correlation coefficients were calculated across the 17 cadavers (kidney vs. liver, brain vs. liver, and brain vs. kidney). Regions yielding an inter-tissue correlation of R^2^ > 0.50 (R > 0.71) were considered positive for CoRSIV[6].

Monocyte DNA methylation in CoRSIVs and Controls

Methylation data from CD14-positive, CD16-negative classical monocytes, in bigwig format, from 6 different individuals (C005PS, S000RD, C000S5, C0010K, C001UY, C004SQ) were imported directly into the UCSC genome browser from the Blueprint Epigenome data repository[8]. BED files containing CoRSIV and control coordinates were uploaded into the UCSC genome browser as custom tracks. The GENCODE v29 Comprehensive Transcript Set track was used to display genes overlaps on the browser. Regions containing CoRSIVs or controls were zoomed in to display methylation data within those regions. Regions were highlighted using the UCSC genome browser’s highlight feature for easier visualization.

Validation of DNA methylation in twin (fingernail-bloodspot) samples

As part of an ongoing study into periconceptional effects on the establishment of methylation at CpG sites, fingernail samples were obtained from neonatal twins born. In brief, the parents of approximately 100 neonatal twin pairs were approached in postpartum recovery rooms or the neonatal intensive care unit at Texas Children’s Hospital in Houston, Texas. *Inclusion criteria* for infants’ participation in the study included being from a twin birth with available pregnancy medical records, and with both a mother >= 18 years (required for study assent in Texas) and a predominantly English-speaking parent. *Exclusion criteria* included infants from higher-order multiple pregnancies, infants from a pregnancy with twin-to-twin transfusion syndrome, and infants with major congenital anomalies (assessed by medical records and parent-report at the time of recruitment). From the start of the study in October 2016 to the time of publication, the parents of approximately 100 twin pairs have been approached for participation. Written, informed consent has been obtained from 33 twin pairs who have met inclusion criteria. Fingernails samples were successfully collected by nurses at Texas Children’s Hospital using a standard safety nail clipper from 48 neonates (23 twin pairs, plus 2 incomplete pairs). Of these infants, enough tissue for DNA isolation and bisulphite sequencing was collected for 32 neonates who provided data for the current analyses. This protocol was approved by the Institutional Review Board at Baylor College of Medicine (protocol #H-37359).

Overlap of CoRSIVs with Blood DMRs (Using Bcell and Neutrophil)

To evaluate the effect of blood contamination six whole genome bisulfite sequencing datasets from BLUEPRINT epigenome database [8] were downloaded for Bcell (GSM2324434, GSM2324435, GSM2324436) and Neutrophils (GSM2325070, GSM2325071, GSM2325072) tissues in three individuals [9]. 59639 Differentially Methylated Regions (DMRs) were identified using DSS R-package (P < 0.01). We hypothesized that if 30 tissues used in the CoRSIV screening approach is not contaminated with blood, significantly low number of the 9926 CoRSIVs should overlap with (Bcell-Neutrophil) DMRs. To calculate the overlap, we used BEDTools intersect software with parameter (-F) set to 1 (Complete overlap). A χ^2^ test was used to determine significance.

Validation of CoRSIVs using 4 brain regions and blood based database.

To validate CoRSIVs using a larger matched multi-tissue DNA methylation data, we used a comparison of 4 brain regions and blood [10]. CpG sites characterized by highly tissue-specific levels of DNA methylation (paired t-test P < 0.00001) but strong evidence for interindividual co-variation (r^2^ > 0.5) consists of 1,813 sites. Using fisher’s exact test, we calculated the significance of overlap with 1,693 hm450k CpG sites found within CoRSIVs (Table S13). As the background set for fisher exact test, of all hm450k probes were considered.

Selecting Control Regions

CoRSIVs are comprised of strings of 100 bp bins separated by at most 100 bp. We preprocessed the overall set of genomic 100 bp CpG bins, such that for each chromosome ***c*** and each 100 bp bin ***b*** we determined the maximum number of successive bins separated by at most 100 bp starting at bin ***b***. For a CoRSIV on chromosome ***c***, containing ***n*** CpG bins, of overall genomic size ***s***, and overall number of covered CpGs ***g***, we selected a corresponding control region by first matching the chromosome ***c*** then randomly choosing a CpG bin ***b*** starting a cluster of ***n*** successive 100 basepair bins separated by at most 100 basepairs. We set a target relative error ***e*** of 10%, and verified that the control matches

- the overall CoRSIV genomic size ***s*** within a relative error of ***e***%
- the overall number of CpGs ***g*** in the CoRSIV within a relative error of ***e***%.

If the relative error target was not achieved for either genomic size or CpG number, then another attempt to select a naïve control was made. If after 100 attempts the relative error for size and number of CpGs was still not achieved, the target relative error for the naïve control would be doubled (eg 10% to 20%, or 20% to 40% etc), and the selection process resumed. This process was then applied for each CoRSIV and therefore generated an equal number of matched control regions.

Selecting tDMR Controls

We used DSS[11] to identify tissue-wise differentially methylated CpG loci (tDMLs) with a minimum read depth of 10x per library using the general experimental design with default smoothing parameters to make pairwise comparisons between different tissue types. DMLs were adjusted for genome-wide significance using an FDR cutoff of 0.05. Tissue differentially methylated regions (tDMRs) were called with a significance threshold of FDR<0.05 and absolute delta >= 20%.

For each comparison (i.e. thyroid vs. heart, thyroid vs. brain, brain vs. heart), we first identified the 100 bp CpG bins overlapping with their respective tDMRs, then preprocessed the filtered set of genomic 100 bp CpG bins, such that for each chromosome ***c*** and each 100 bp bins ***b*** we determined the maximum number of successive bins separated by at most 100 bp starting at bin ***b***.

For a CoRSIV on chromosome ***c***, containing ***n*** CpG bins, of overall genomic size ***s***, and overall number of covered CpGs ***g***, we first selected the tissue pair comparison, such that each comparison was equally represented in the final set of tDMR controls. We set a target relative error ***e*** of 10%, and verified that the control matches

- the overall CoRSIV genomic size ***s*** within a relative error of ***e***%
- the overall number of CpGs ***g*** in the CoRSIV within a relative error of ***e***%.

If the relative error target was not achieved for either genomic size or CpG number, then another attempt to select a naïve control was made. If after 100 attempts the relative error for size and number of CpGs was still not achieved, the target relative error for the naïve control would be doubled (eg 10% to 20%, or 20% to 40% etc), and the selection process resumed.

Overlap of CoRSIVs in Sub-telomeric Regions

Enrichment of CoRSIVs at sub-telomeric regions was calculated as previously described[6]. Sub-telomeric regions were defined as the 1 Mb region interior to each telomere. The number of 100 bp CoRSIV bins was counted and compared to the full set of 100 bp bins that overlapped the sub-telomeric regions of each chromosome. A χ^2^ test was used to determine significance.

Overlap of CoRSIVs in Repetitive Elements and CpG Islands

Repetitive element and CpG island (CGI) annotations for the hg38 genome were downloaded from the UCSC genome browser’s table browser (https://genome.ucsc.edu/cgi-bin/hgTables). These features were filtered by type: LINE, SINE, Endogenous retroviruses (ERVs), and CpG islands (CGI). Bedtools[12] was used to quantify the number of CoRSIV regions that overlapped each of these features compared to control regions. A χ^2^ test was used to determine significance.

Overlap of CoRSIVs in Transcription Factor Binding Sites

Annotated transcription factor binding sites were downloaded from the ORegAnno database[13]. Bedtools[12] was used to quantify the number of CoRSIV regions and control regions that overlapped the known transcription factor binding sites. A χ^2^ test was used to determine significance.

Assessment of Epigenome States Enrichment in CoRSIVs

We downloaded the 15 epigenome states defined based on 127 epigenomes published by the NIH Epigenome Roadmap[14] and the Encode consortia using the Washington University Epigenome Browser[15]. We used the UCSC liftOver tool to convert coordinates for CoRSIVs, controls, and tDMR regions from hg38 to hg19. We determined overlap with each epigenome state and in each of the 127 epigenomes using BEDTOOLS[12]. Enrichment of CoRSIVs over Controls or tDMRs was assessed using Fisher’s exact test, with significance achieved for P < 0.05. Odd-ratio visualizations were generated using the Python scientific library.

Evaluation of Enrichment of SuperCoRSIVs within TADs

We downloaded a compendium of TADs in multiple human tissues[16] and evaluated the likelihood of SuperCoRSIVs residing completely within TADs. Using bedtools software package we computed the number of SuperCoRSIVs and control regions that completely reside within the TAD boundaries for each of the 10 tissues. We did a paired t-test to evaluate the significance of the difference in enrichment for SuperCoRSIVs vs. control regions.

SuperCoRSIV Overlap with CTCF Binding Sites

CTCF binding sites were obtained from the ORegAnno database. Bedtools was used to calculate the overlap between CTCF binding sites and SuperCoRSIV regions as well as the SuperControl regions. SuperCoRSIV regions included all the genomic regions between the first and last CoRSIV making up the SuperCoRSIV. A χ^2^ test was used to determine significance.

CoRSIVs overlapping ENCODE blacklist regions

We downloaded black listed regions in bed format from ENCODE database using the following link. <http://mitra.stanford.edu/kundaje/akundaje/release/blacklists/hg38-human/hg38.blacklist.bed.gz>. CoRSIV regions were intersected with black list regions using bedtools software (at least 1bp overlap).

mQTL Analysis Using Genotype Data from 10 Gtex Donors

Data on SNV genotype were downloaded from GTeX. Of all SNVs located within 10 kb of each CoRSIV, we retained only those with a minor allele frequency of ≥ 0.2, and further considered only those SNVs in which all three genotypes are represented among the 10 GTEx donors. We performed linear regression to examine the correlation between individual methylation levels at each CoRSIV and the genotypes of SNVs in *cis*. For all informative SNVs within 10kb of each CoRSIV, we assigned each genotype a numeric value corresponding to the number of minor alleles: AA = 0, Aa = 1, aa = 2. In the linear regression, the coefficient β of the function reflects the additive effect of one minor allele, R^2^ reflects the goodness of fit to the linear function, and the p value corrected for multiple testing using Benjamini-Hochberg method (False Discovery Rate) reflects the significance. All CoRSIV-SNV pairs with a regression coefficient β ≥ 10, an R^2^ ≥ 0.5, and a FDR < 5% were considered positive for mQTL. MatrixEQTL was used for performing linear regression.

Evaluating Pairwise Associations Between Genotype and Methylation

Using SNV genotype data downloaded from GTeX, we considered all common SNVs (minor allele frequency ≥0.2) within 10 kb of each CoRSIV. SNV genotypes were converted to numerical values as described above. For all pairwise comparisons among the 10 GTEx donors, we calculated the interindividual difference in average methylation at each CoRSIV and the average interindividual difference in associated SNV values. Linear regression was used to calculate the Pearson R^2^ correlation of pairwise differences in CoRSIV methylation vs. pairwise differences in average neighborhood SNV value.

Analysis of published mQTL data for HM450 probes within CoRSIVs

Results from multiple mQTL analyses were obtained[17-21]. Probes from each dataset were filtered using the same p-value threshold of significance as the respective authors used. Significant mQTL probes were then filtered for the probes within CoRSIV regions, creating a list of probes which exhibited mQTL and are also found in CoRSIV regions. For CoRSIVs containing multiple mQTL probes, the probe with the lowest p-value was used.

Evaluate the association of Copy Number Variation (CNV) in CoRSIV regions

Population CNV map in tab delimited format was downloaded from the DECIPHER database[22], using the following link: [*https://decipher.sanger.ac.uk/files/downloads/population_cnv.txt.gz*](https://decipher.sanger.ac.uk/files/downloads/population_cnv.txt.gz). The population CNV regions were mapped to hg38 genome using LiftOver software available in UCSC Genome browser. CoRSIV regions were intersected with population CNV regions (at least 1 bp overlap) using BEDTOOLS intersect software. Odds ratios were calculated using fisher’s exact test.

To find the association of the effect of CNVs (Deletions or Duplications) in CoRSIV methylation. We extracted the read depth of each CoRSIV region from the 30 samples. “Samtools bedcov” software was used to count total number of bases overlapping each CoRSIV region and divided by CoRSIV genomic width to obtain the read depth for each sample. The average read depth and average methylation across three tissues for each CoRSIV region for 10 individuals were obtained. The spearman correlation coefficient between avg. methylation levels and avg. read depth was calculate across the 10 individuals for all 9926 CoRSIVs.

Comparing Methylation R^2^ with Linkage Disequilibrium (LD) R^2^ at CoRSIVs using 1000Genomes data

We compared the LD decay in CoRSIV regions with the decay of methylation R^2^, similar to a previously published method[23]. For this analysis, we considered all CoRSIVs with at least one SNV (MAF ≥ 0.2) located within the CoRSIV (we refer to this as the ‘index CoRSIV’).

For each CoRSIV, following steps were carried out;

1. Compute methylation R^2^

Using all methylation bins within +/- 20 kb from the index CoRSIV bins, we computed R^2^ and distance between all pairs of bins where one bin is inside the CoRSIV. This computation is done for all pairs of bins.

1. Compute LD

In the case of SNVs, we first downloaded the genotype data in VCF format from the 1000 Genomes project[24]. These VCF files were filtered to separate the genotypes of CEU (Northern Europeans from Utah) individuals. Using “VCFtools” software package we computed the LD between all pairs of SNVs (MAF ≥ 0.2) [25]. We used LD values from all pairs of SNVs where each pair has one SNV resides within the index CoRSIV and other SNV is within +/- 20 kb.

1. Evaluate association between methylation correlation and LD

The +/- 20 kb region flanking the CoRSIV is split in to 100 bp windows. Median of the methylation R^2^ values which falls within each 100bp window is calculated and stored in to a vector$(i)$. Similarly, median of LD values which falls within each 100bp window is calculated and stored in to a vector$(j)$. Then spearman correlation coefficient P-value between vector$\left( i \right)$ and vector$(j)$ is computed to determine a significant association. Only the positive spearman correlation coefficients and P values were considered to evaluate the association between methylation and LD.

CoRSIVs with significant association (P value ≤ 0.05) were considered as evidence for methylated regions that are under genetic influence. While we recognize that this P value is not strictly valid because of the non-independence of methylation values and SNV genotypes in neighboring bins, we view this as a conservative error as it would only tend to increase the statistical significance of the methylation decay vs. LD decay association.

CoRSIV methylation and gene expression correlation

Methylation and gene expression data published by Grundberg et al.[19] were downloaded from the European Bioinformatic Institute ArrayExpress, accession IDs E-TABM-1140 and E-MTAB-1866. The genomic location of each CpG probe on the HM450 array provided by Illumina (hg19 coordinates) was converted to hg38 using the LiftOver command line tool on the UCSC genome browser. HM450 data from adipose tissue of 649 individuals were filtered to retain probes that were found in CoRSIVs. This results in 1,658 probes located in 819 CoRSIVs. For CoRSIVs with multiple probes, the mean methylation value of the probes was used.

CoRSIVs were associated to genes and one or more features: promoter, gene body, 3’ regions if the CoRSIV overlapped the transcription start site (TSS) +/- 3kb, anywhere between the TSS and transcription end site (TES), or the TES +/- 3kb respectively. Note that it is possible for one CoRSIV to be associated with multiple features of the same gene. Using this method, 645 out of 819 CoRSIVs were associated with at least one gene.

Gene expression data from the same 649 individuals was obtained for three different tissues: adipose, skin, and lymphoblastoid cell lines (LCL)[19]. Spearman correlations between CoRSIV methylation and gene expression data were calculated for each gene with both methylation and expression data available. These P values were adjusted for multiple testing using Benjamini/Hochberg false discovery rate. The correlation was considered significant if the adjusted P value was < 0.05.

Enrichment of EWASdb HM450k probes and diseases in CoRSIV regions

We downloaded the single marker database from EWASdb[26], an epigenome-wide association database combining the results of 1319 EWAS studies associated with 302 diseases. This database consists of 18,538,029 hm450k probes that are differentially methylated between case-control datasets (t-test, P < 1x10^-7^). We overlapped these probes with CoRSIV, control, and tDMR regions and counted the number of disease related EWAS. Pearson’s Chi-squared test was used to determine the significance of the associations for CoRSIV vs. control regions and CoRSIV vs. tDMR regions. To explore which diseases are mostly associated with CoRSIVs,controls or tDMRs we used the following approach. First, the database was filtered by number of hm450k probes associated with each EWAS, with more than 5000 significant probes contributed to the database were filtered out to eliminate noise from non-significant associations. Then, the list of diseases in were separated to two categories; cancer related diseases and non-cancer related diseases. Heatmaps were generated to visualize the significance of association P values of CoRSIV, Control, and tDMR probes with diseases in the two categories. Maximum -log10(P value) and number of probes associated with each disease were plotted in a scatter plot and a linear model was used to fit a line.

CoRSIV association with periconceptional environment

In a series of studies in a subsistence-farming community in rural Gambia we have exploited a natural experiment in which an extreme annual pattern of rainy and dry seasons drives significant seasonal patterns in dietary intake and of one-carbon metabolites measured in plasma[7, 27]. We previously observed associations between the season in which an individual is conceived and DNA methylation in blood at sites exhibiting SIV[6, 7, 28-30] thus providing human evidence to support prior observations in mouse that periconceptional nutrition influences methylation at sites of SIV[31, 32]. We explored the potential influence of season of conception on CoRSIV methylation in peripheral blood DNA from 233 Gambian 2 year olds, measured on the HM450 array[29]. After filtering for quality control, 1,101 CpGs mapping to CoRSIVs, 2,213 to negative controls, and 2,823 to tDMRs were considered for analysis. The association between methylation and date of conception was modelled using Fourier regression analysis[33, 34]. Fourier regression models included up to two pairs of Fourier terms, with the number of terms determined by likelihood ratio test (LRT). All models were adjusted for sex and major principal components derived from unsupervised PCA of the methylation data associated with technical artefacts and differences in estimated cell composition[35]. Fourier regression revealed significant seasonal variation at 77 CoRSIV CpGs, 71 negative control CpGs, and 112 tDMR CpGs, respectively (FDR<20%, LRT Fourier model with 1 pair of Fourier terms vs baseline model). Enrichment of seasonal effects at CoRSIVs, negative controls and tDMRs was determined by analyzing the proportion of significant CpGs in each group, compared to the same proportion across all 6,137 CpGs belonging to all 3 groups using Fisher’s Exact Test. Date of conception methylation maxima and minima for each CpG were determined from the fitted models using significant Fourier terms only. The ‘seasonal amplitude’ (Figure S12B) was defined as the absolute difference between the predicted peak and nadir of (date of conception-related) methylation.

Assessment of gene-disease associations based on Pubmed

To mine PubMed entries for gene/disease associations we used the Pubtator framework using a three step strategy[36]. 1) For a human gene *hGene*, we determined the orthologs in other species represented as NCBI gene identifiers, *oGene_1_*, *oGene_2_*, .., *oGene_m_*. 2) We determined all Pubmed entries referring to either the *hGene* or any of its orthologs *oGene_1_*, *oGene_2_*, .., *oGene_m_*, obtaining a set of Pubmed identifies *pmid_1_, pmid_2_, .., pmid_p_* 3) Finally, we compiled the list of all diseases referenced in *pmid_1_, pmid_2_, .., pmid_p_* denoted as *disease_1,_ disease_2_, …, disease_d ._* Pubtator utilizes the Medical Subject Headings (MESH, https://www.nlm.nih.gov/mesh/) nomenclature.

For each disease *disease_i_* we determined the CoRSIV-associated human genes related to disease based on Pubtator. For convenience of the reader, we selected the top 100 diseases sorted by the overall number of associated human genes, then we summarized them using MESH codes provided by the MESH repository.

GO analysis

Hypo- and hyper-methylated tDMRs for each pairwise comparison were lifted over to Hg19 and separately associated with cis-regulatory regions by GREAT v3.09[37], and the top 8 enriched Gene Ontology (GO) process terms for each were reported. Complete set of genes were used as the background set for the enrichment analysis.

**References**

1. GTEx Consortium, *The Genotype-Tissue Expression (GTEx) project.* Nat Genet, 2013. **45**(6): p. 580-5.

2. Kunde-Ramamoorthy, G., et al., *Comparison and quantitative verification of mapping algorithms for whole-genome bisulfite sequencing.* Nucleic Acids Res, 2014. **42**(6): p. e43.

3. Liu, Y., et al., *Bis-SNP: Combined DNA methylation and SNP calling for Bisulfite-seq data.* Genome Biol, 2012. **13**(7): p. R61.

4. Snedecor, G., Cochran, WG, *Statistical Methods*. 8th ed. 1989, Ames, Iowa: Iowa State University Press.

5. Shen, L., et al., *Optimizing annealing temperature overcomes bias in bisulfite PCR methylation analysis.* Biotechniques, 2007. **42**(1): p. 48-58.

6. Silver, M.J., et al., *Independent genomewide screens identify the tumor suppressor VTRNA2-1 as a human epiallele responsive to periconceptional environment.* Genome Biol, 2015. **16**: p. 118.

7. Dominguez-Salas, P., et al., *Maternal nutrition at conception modulates DNA methylation of human metastable epialleles.* Nat Commun, 2014. **5**: p. 3746.

8. Fernandez, J.M., et al., *The BLUEPRINT Data Analysis Portal.* Cell Syst, 2016. **3**(5): p. 491-495 e5.

9. Farlik, M., et al., *DNA Methylation Dynamics of Human Hematopoietic Stem Cell Differentiation.* Cell Stem Cell, 2016. **19**(6): p. 808-822.

10. Hannon, E., et al., *Interindividual methylomic variation across blood, cortex, and cerebellum: implications for epigenetic studies of neurological and neuropsychiatric phenotypes.* Epigenetics, 2015. **10**(11): p. 1024-32.

11. Park, Y. and H. Wu, *Differential methylation analysis for BS-seq data under general experimental design.* Bioinformatics, 2016. **32**(10): p. 1446-53.

12. Quinlan, A.R., *BEDTools: The Swiss-Army Tool for Genome Feature Analysis.* Curr Protoc Bioinformatics, 2014. **47**: p. 11 12 1-34.

13. Lesurf, R., et al., *ORegAnno 3.0: a community-driven resource for curated regulatory annotation.* Nucleic Acids Res, 2016. **44**(D1): p. D126-32.

14. Roadmap Epigenomics, C., et al., *Integrative analysis of 111 reference human epigenomes.* Nature, 2015. **518**(7539): p. 317-30.

15. Zhou, X., et al., *The Human Epigenome Browser at Washington University.* Nat Methods, 2011. **8**(12): p. 989-90.

16. Schmitt, A.D., et al., *A Compendium of Chromatin Contact Maps Reveals Spatially Active Regions in the Human Genome.* Cell Rep, 2016. **17**(8): p. 2042-2059.

17. Bonder, M.J., et al., *Disease variants alter transcription factor levels and methylation of their binding sites.* Nat Genet, 2017. **49**(1): p. 131-138.

18. Gaunt, T.R., et al., *Systematic identification of genetic influences on methylation across the human life course.* Genome biology, 2016. **17**(1): p. 61.

19. Grundberg, E., et al., *Global analysis of DNA methylation variation in adipose tissue from twins reveals links to disease-associated variants in distal regulatory elements.* Am J Hum Genet, 2013. **93**(5): p. 876-90.

20. Ng, B., et al., *An xQTL map integrates the genetic architecture of the human brain's transcriptome and epigenome.* Nature neuroscience, 2017. **20**(10): p. 1418.

21. Shi, J., et al., *Characterizing the genetic basis of methylome diversity in histologically normal human lung tissue.* Nature communications, 2014. **5**: p. 3365.

22. Firth, H.V., et al., *DECIPHER: Database of Chromosomal Imbalance and Phenotype in Humans Using Ensembl Resources.* Am J Hum Genet, 2009. **84**(4): p. 524-33.

23. Schmitz, R.J., et al., *Patterns of population epigenomic diversity.* Nature, 2013. **495**(7440): p. 193-8.

24. Genomes Project, C., et al., *A global reference for human genetic variation.* Nature, 2015. **526**(7571): p. 68-74.

25. Danecek, P., et al., *The variant call format and VCFtools.* Bioinformatics, 2011. **27**(15): p. 2156-8.

26. Liu, D., et al., *EWASdb: epigenome-wide association study database.* Nucleic Acids Res, 2018.

27. Dominguez-Salas, P., et al., *DNA methylation potential: dietary intake and blood concentrations of one-carbon metabolites and cofactors in rural African women.* Am J Clin Nutr, 2013. **97**(6): p. 1217-27.

28. Kuhnen, P., et al., *Interindividual Variation in DNA Methylation at a Putative POMC Metastable Epiallele Is Associated with Obesity.* Cell Metab, 2016. **24**(3): p. 502-9.

29. Van Baak, T.E., et al., *Epigenetic supersimilarity of monozygotic twin pairs.* Genome Biol, 2018. **19**(1): p. 2.

30. Waterland, R.A., et al., *Season of conception in rural gambia affects DNA methylation at putative human metastable epialleles.* PLoS Genet, 2010. **6**(12): p. e1001252.

31. Waterland, R.A., et al., *Maternal methyl supplements increase offspring DNA methylation at Axin fused.* Genesis, 2006. **44**(9): p. 401-6.

32. Waterland, R.A. and R.L. Jirtle, *Transposable elements: targets for early nutritional effects on epigenetic gene regulation.* Mol Cell Biol, 2003. **23**(15): p. 5293-300.

33. Fulford, A.J., P. Rayco-Solon, and A.M. Prentice, *Statistical modelling of the seasonality of preterm delivery and intrauterine growth restriction in rural Gambia.* Paediatr Perinat Epidemiol, 2006. **20**(3): p. 251-9.

34. Nabwera, H.M., et al., *Growth faltering in rural Gambian children after four decades of interventions: a retrospective cohort study.* Lancet Glob Health, 2017. **5**(2): p. e208-e216.

35. Jaffe, A.E. and R.A. Irizarry, *Accounting for cellular heterogeneity is critical in epigenome-wide association studies.* Genome Biol, 2014. **15**(2): p. R31.

36. Wei, C.H., H.Y. Kao, and Z. Lu, *PubTator: a web-based text mining tool for assisting biocuration.* Nucleic Acids Res, 2013. **41**(Web Server issue): p. W518-22.

37. McLean, C.Y., et al., *GREAT improves functional interpretation of cis-regulatory regions.* Nat Biotechnol, 2010. **28**(5): p. 495-501.
