# Supplementary material for: A genomic atlas of systemic interindividual epigenetic variation in humans
Source: Genome Biol. 2019 Jun 3;20:105. doi: 10.1186/s13059-019-1708-1 (PMC6545702; doi:10.1186/s13059-019-1708-1)
Supplement: Supplementary file 4 — Supplementary Figures. (DOCX 15207 kb) [file 13059_2019_1708_MOESM4_ESM.docx]

Supplementary Figures for

**A Genomic Atlas of Systemic Interindividual Epigenetic Variation in Humans**

Chathura J. Gunasekara, C. Anthony Scott, Eleonora Laritsky, Maria S. Baker, Harry MacKay, Jack D. Duryea, Noah J. Kessler, Garrett Hellenthal, Alexis C. Wood, Kelly R. Hodges, Manisha Gandhi, Amy B. Hair, Matt J. Silver, Sophie E. Moore, Andrew M. Prentice, Yumei Li, Rui Chen, Cristian Coarfa*, Robert A. Waterland^*^

^*^Correspondence to: [waterland@bcm.edu](mailto:waterland@bcm.edu) or [coarfa@bcm.edu](mailto:coarfa@bcm.edu)

**This file includes:**

Supplementary Figures S1 to S16

References cited in supplementary figure legends

**Other Supplementary Material for this manuscript includes:**

- Supplementary Tables S1 to S19 (Except Table S2) (Additional File 1)
- Supplementary Table S2 (Additional File 2)
- Supplementary methods (Additional File 3)

**
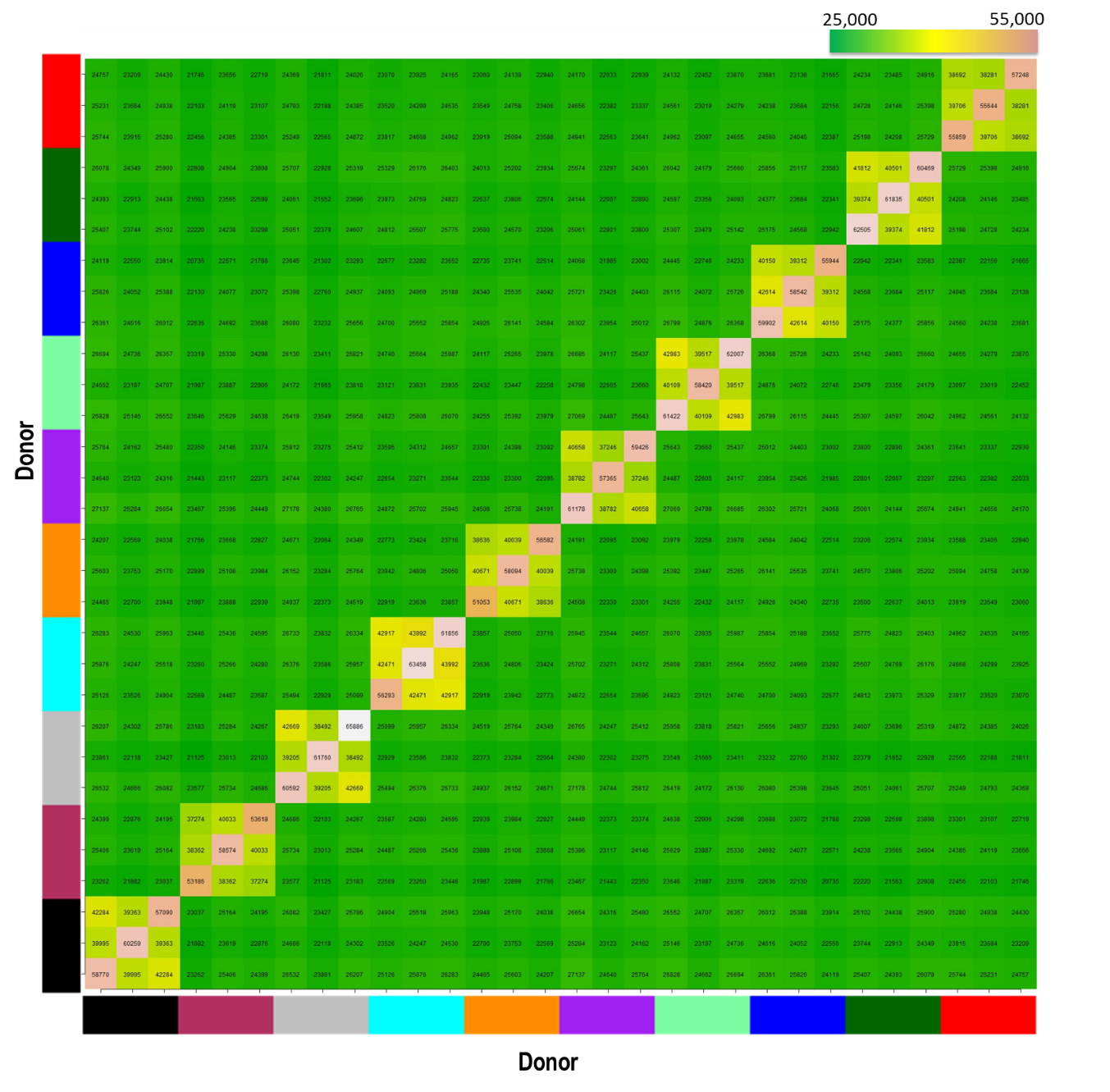
**

**Fig. S1: Results from SNP-calling the 30 libraries in Chr19 to ensure that different tissue samples were properly assigned to the same individual.** All pair-wise tissues were compared to count the number of SNPs common in both tissues. These counts were then assembled in to a matrix. The three tissues from each donor cluster together.

**Fig. S2: CoRSIV characteristics and enrichment of genomic features.** **a,** Results from permutation test to evaluate if CoRSIVs with few CpGs can occur randomly. **b,** Cutoffs set to filter out robust epigenetic varients (nCPG >=5, IIR >=20). **c,** Distribution of the permutation P values of 9926 CoRSIVs. **d,** Distribution of ITC of CoRSIVs. **e,** Analysis of epigenomic features relative to tDMR regions. **f,** (top) Distribution of SuperCoRSIV sizes, (bottom) enrichment of SuperCoRSIVs completely within TADs compared to control regions. **g,** Enrichment of CTFC binding sites in SuperCoRSIVs vs Controls (χ^2^ test P = 0.003). **h,** Enrichment/depletion of CoRSIVs in specific transcription binding sites compared to control and tDMR regions. **i.** Odds ratios for the overlap of CoRSIVs, Controls and tDMRs in Genomic Evolutionary Rate Profiling (GERP) regions. (Fisher’s Exact Test, CoRSIVs vs Control : P = 8.24e-118, CoRSIVs vs tDMRs: P = 1e-200)

**
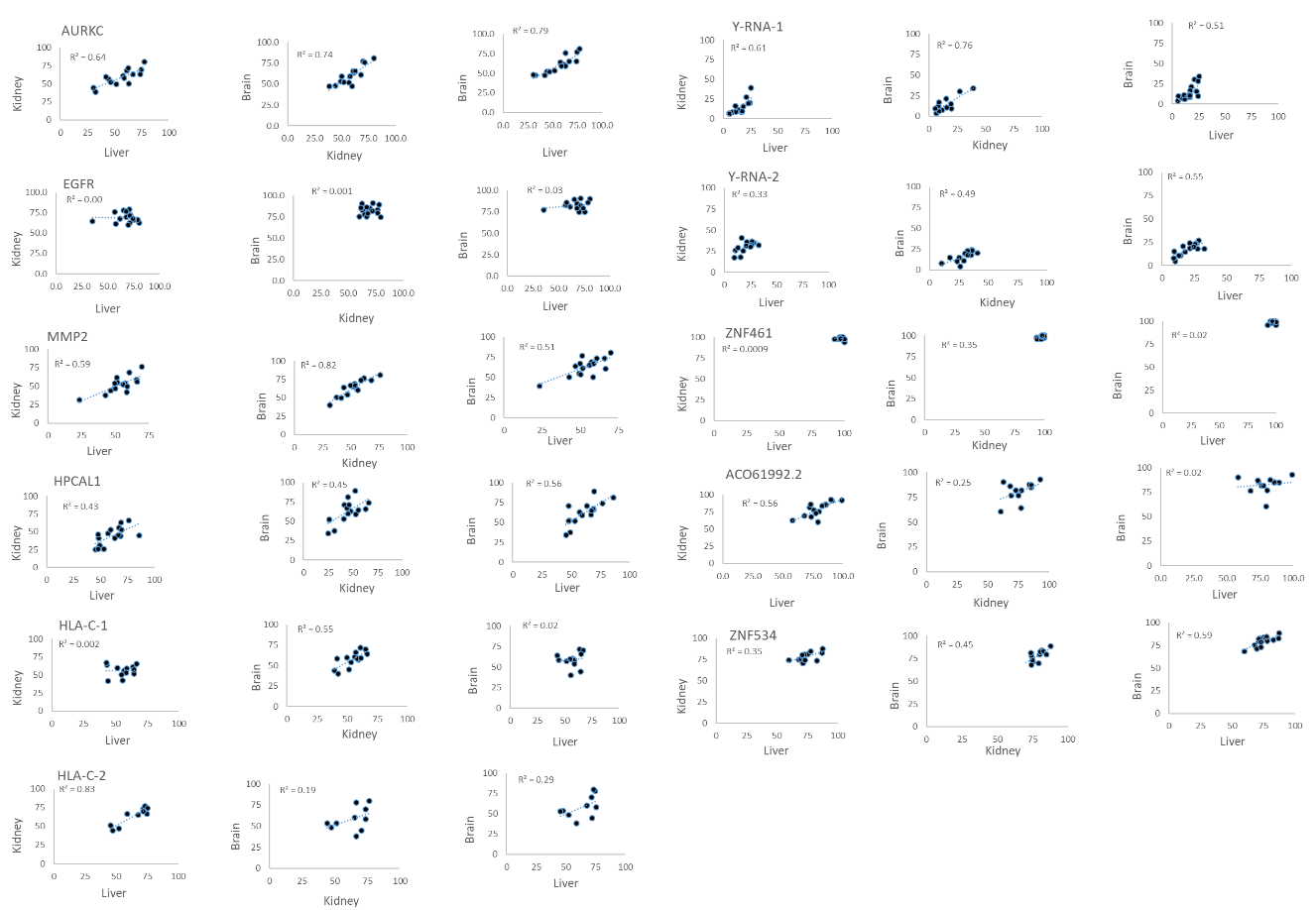
**

**Fig. S3: Validation of CoRSIV regions**. Results from quantitative analysis of selected CoRSIVs by bisulfite pyrosequencing across endodermal (liver), mesodermal (kidney), and ectodermal (brain) tissue in 17 Vietnamese cadavers. Of 11 regions evaluated, 9 (82%) showed a correlation R^2^ > 0.5 in at least one inter-tissue comparison, indicative of SIV. *EGFR* and *ZNF461* showed too little interindividual variation to qualify as CoRSIVs in this Asian sample.

**Fig. S4: Interindividual variation at CoRSIV/Control regions in purified monocyte populations from different donors. a,** CoRSIV regions (first track, blue highlight) show substantial interindividual variation at in six CD14-positive, CD16-negative classical monocytes samples from different donors (Blueprint Epigenome samples C005PS, S000RD, C000S5, C0010K, C001UY, C004SQ; red tracks, track 3-8). Overlap with neighboring genes is shown; arrows heads indicate gene direction (second track). **b,** Control regions (first track, blue highlight) show minimal interindividual variation at control regions in six CD14-positive, CD16-negative classical monocytes samples from different donors (Blueprint Epigenome samples C005PS, S000RD, C000S5, C0010K, C001UY, C004SQ; red tracks, tracks 3-8). Overlap with neighboring genes is shown; arrows heads indicate gene direction (second track). **c.** Comparison of Inter-Individual Range (IIR) in Blueprint monocite DNA methylation in CoRSIVs vs. controls. The histogram shows 1235 CoRSIV regions and 1364 control regions with at least 10 CpGs.

**Fig. S5: Validations of CoRSIV regions using human tissue devoid of blood (Finger nails) and blood spots.** Results from quantitative analysis of selected CoRSIVs by bisulfite pyrosequencing. 20-30 individuals were used for this analysis. Although a CoRSIV in VTRNA2-1gene was not detected in this CoRSIV screen, existance of a SIV region in VTRNA2-1 has been validated (P < 0.05) in two of our previous studies[1]^,^[2].

**Fig. S6: Distribution of methylation levels in CoRSIV regions.** **a,** Density plot represents the methylation levels for each of the 10 individuals in all 9926 CoRSIV regions. **b,** The CoRSIVs were separated in to two categories based on existing mQTL evidence in the literature (Table S14) and density plots were generated separately. The density plots on the left used 557 CoRSIVs with mQTL evidence and density on the plots on the right used 263 CoRSIVs with no mQTL evidence.

**Fig. S7: Characteristics of two CoRSIVs overlapping SPATC1L.** **a,** The two anti-correlated CoRSIVs are located over TSS and TES of SPATC1L gene. **b,** Association analysis (Spearman Correlation) between methylation (Adipose Tissue) and gene expression in three tissues (Adipose, Skin, LCL).

**Fig. S8: Distribution of CoRSIVs in chromosomes. a,** MHC locus of chr6 is densely populated with CoRSIVs as well as genes. **b,** CoRSIV map of the MHC locus. Most CoRSIVs in this region are not correlated with one another. **c,** Pericentromeric region of chr20 is densely populated with CoRSIVs but sparsely populated with genes. **d,** CoRSIV map of the chr20 pericentromeric region. Most CoRSIVs in this region are strongly correlated with one another, indicating consistent individual methylation across the entire >2Mb region.

**Fig. S9: Distribution of CoRSIVs across each human autosome.**

**Fig. S10: Average read depth diagrams for 30 methylomes** in **a**, chr 20 start(1Mb), end (1Mb) **b,** chr 6 start (1Mb), MHC CoRSIV region, end (1Mb), peak CoRSIV region.

**Fig. S11: tDMR detection, control regions and tDMR controls.** **a,** Control regions are matched to CoRSIVs on the basis of CpGs per region (left, red) and size (right, blue). **b,** Data on all tDMRs identified, showing distributions of DMR size (left) and magnitude of differential methylation (right) for heart vs. thyroid (top), brain vs. thyroid (middle), and brain vs. heart (bottom). **c,** The tDMR set used in analyses is matched to the CoRSIVs on the basis of CpGs per region (left, red) and size (right, blue).

**Fig. S12: Associations of CoRSIVs with periconceptional environment and disease.** **a,** Predicted change in methylation with month of conception at CoRSIVs (red, n=77 CpGs), negative controls (green, n=71) and tDMRs (blue, n=112). Only loci with significant seasonal variation (FDR<20%; likelihood ratio test, are shown. Y-axis represents seasonal change relative to annual mean in adjusted models. **b,** Distributions of modeled ‘seasonal amplitude’ for CoRSIV, negative control and tDMR CpGs on the HM450. Seasonal amplitude is defined as the absolute distance between the peak and nadir of (date of conception-related) methylation (see inset for single CpG). Only loci with significant seasonal variation (FDR<20%; likelihood ratio test are shown. Methylation is significantly greater for CoRSIVs vs negative controls (median CoRSIV amplitude = 4.9%, negative controls = 1.4%; Wilcoxon rank-sum test P<2.2x10^-16^); and for CoRSIVs vs tDMRs (mean tDMR amplitude = 2.1%; P<5.3x10^-14^). **c,** Results from PubMed mining using PubTator software. The proportion of CoRSIVs overlapping genes associated with diseases shown in the pie chart. **d,** Distribution of gene body size to compare the association of CoRSIVs, controls, and tDMRs. **e,** The number of HM450K probes and number of EWAS studies overlapping CoRSIVs, controls, and tDMRs. Compared to control and tDMR probes, CoRSIV probes are 37% (χ^2^ test , P=2.2x10^-4^) and 57% (χ^2^ test , P=6.5x10^-8^) are associated with EWAS related to diseases.

**Fig. S13: Evaluating association between methylation and gene expression.** **a,** P-value distribution for the spearman correlation between gene expression and DNA methylation. **b,** Venn diagram illustration counts of significant associations shared among different tissues in control and tDMR control regions. Unlike at CoRSIVs, significant correlations (P < 0.05) between methylation in adipose tissue and expression in adipose tissue are generally not maintained between methylation in adipose tissue and expression in skin or lymphoblastoid cell lines.

**Fig. S14: Visualization of association between significant hm450k probes in EWASdb and cancer related diseases.** The probes are arranged by chromosomal position from chromosome 1 to chromosome 22 **a,** Heatmap for P values of significant CoRSIV probes in each disease. **b,** Heatmap for P values of significant Control probes in each disease. **c,** Heatmap for P values of significant tDMR probes in each disease. **d,** Scatter plot shows maximum -log10 (P value) for each disease in the x-axis and with number of significant probes associated with each disease on the y-axis. A linear model was used to fit the data and 95% confidences intervals are shown in gray color.

**Fig. S15: Visualization of association between significant hm450k probes in EWASdb and non-cancer related diseases.** The probes are arranged by chromosomal position from chromosome 1 to chromosome 22 **a,** Heatmap for P values of significant CoRSIV probes in each disease. **b,** Heatmap for P values of significant Control probes in each disease. **c,** Heatmap for P values of significant tDMR probes in each disease. **d,** Scatter plot shows maximum -log10 (P value) for each disease in the x-axis and with number of significant probes associated with each disease on the y-axis. A linear model was used to fit the data and 95% confidences intervals are shown in gray color.

**Fig. S16: Results from the analysis of CoRSIVs using GREAT software tool.** **a,** Biological Function, **b,** Biological Process, and **c,** Cell Component categories significantly enriched (FDR<0.05) in CoRSIV-associated genes. **d,** Disease ontology terms significantly enriched (FDR<0.05) in CoRSIV-associated genes.

**References cited in supplementary figure legends**

1. Silver, M.J., et al., *Independent genomewide screens identify the tumor suppressor VTRNA2-1 as a human epiallele responsive to periconceptional environment.* Genome Biol, 2015. **16**: p. 118.

2. Van Baak, T.E., et al., *Epigenetic supersimilarity of monozygotic twin pairs.* Genome Biol, 2018. **19**(1): p. 2.
